# Antibody Response to SARS-CoV-2 Membrane Protein in Patients of the Acute and Convalescent Phase of COVID-19

**DOI:** 10.3389/fimmu.2021.679841

**Published:** 2021-08-04

**Authors:** Philipp Jörrißen, Paula Schütz, Matthias Weiand, Richard Vollenberg, Inga Marie Schrempf, Kevin Ochs, Christopher Frömmel, Phil-Robin Tepasse, Hartmut Schmidt, Andree Zibert

**Affiliations:** Medizinische Klinik B, Universitätsklinikum Münster, Münster, Germany

**Keywords:** SARS-CoV-2, COVID-19, antibodies, ELISA, membrane protein, RBD, immunodominant epitopes, B-cell epitope mapping analyses

## Abstract

Understanding the course of the antibody response directed to individual epitopes of SARS-CoV-2 proteins is crucial for serological assays and establishment of vaccines. Twenty-one synthetic peptides were synthesized that have ten amino acids overlap and cover the complete membrane (M) protein. Plasma samples from 32 patients having acute disease and 30 patients from the convalescent phase were studied. Only peptide M01 (aa 1–20) and to a lesser extent peptide M21 (aa 201–222) showed specific reactivity as compared to historical control plasma samples. Peptide M01 was recognized by IgM- (71.9%) and IgG-specific antibodies (43.8%) during the acute phase as early as day 8 PIO. In a longitudinal analysis, a higher reactivity was observed for the IgM response directed to peptide M01 following day 20 PIO as compared to earlier time points of the acute phase. In the convalescent phase, antibody reactivity to the two M-specific peptides was significantly lower (<30% seropositivity). A fusion protein encoding major parts of RBD also showed higher rates of recognition during acute (50.0%) and lower rates in the convalescent phase (23.3%). Taken together, our results suggest that during the acute phase of COVID-19 antibodies are raised to two linear epitopes of the SARS-CoV-2 M protein, located at the very N- and C-termini, showing almost similar levels of reactivity as immunodominant linear epitopes derived from the spike and nucleocapsid protein. Anti-M is also present in the convalescent phase of COVID-19 patients, however at lower levels, with the N-terminus of the M protein as a preferred target.

## Introduction

Severe acute respiratory syndrome coronavirus 2 (SARS-CoV-2) is the etiological agent of the pandemic coronavirus disease 2019 (COVID-19). SARS-CoV-2 is a member of the betacoronavirus genus and belongs to the class of enveloped viruses having a diameter of ~80–120 nm containing a positive single-stranded RNA genome of about 30 kb (1, 2). The viral RNA of coronaviruses is complexed with four major virus encoded proteins, spike protein (S), small membrane protein (E), membrane protein (M) and basic nucleocapsid protein (N). The structural proteins, expressed in the order S, E, M and N, are encoded by the last third of the viral genome. Several other minor proteins expressed by the viral genome have been implicated. Protein domains of the SARS-CoV-2 S protein have been recently resolved by 3D high resolution analysis (3–6), whereas the 3D structure of the other structural proteins is less understood. The transmembrane S protein, forming a crown-like structure typically found in all coronaviruses, is a helical glycoprotein belonging to the type-I class viral fusion proteins that aggregate to a homotrimer of ~180 kd monomers. The small E protein is highly hydrophobic and postulated to span twice the viral membrane with both N- and C-termini located in the interior of the viral capsid. The M protein of SARS-CoV-2 has 222 amino acids (aa) and is postulated to span three times the viral membrane and has a short, singly-glycosylated N-terminal ectodomain, and a long C-terminal endodomain (7–9).

The spike protein, proteolytically processed by host proteases into two independent domains, named S1 and S2, mediates host cell entry (10, 11). The spike protein directly interacts with human angiotensin-converting enzyme 2 (hACE2) triggering membrane fusion and virus entry (11). The receptor-binding domain (RBD, aa S331-528) of SARS-CoV-2 is located in the S1 subunit and facilitates syncytium formation and viral spreading. RBD is a target of neutralizing antibodies (12). Upon hACE2 binding, the virus enters the cytosol of the host cell followed by fusion of the viral and cellular membranes. The M protein, accounting for the overall shape of the viral envelope, is the most abundant protein of coronaviruses and recruits other structural proteins to the endoplasmatic reticulum (ER)–Golgi intermediate compartment (ERGIC), where the virus assembly and budding of coronaviruses takes place (9, 13). The M protein is thought to stabilize N proteins and promotes completion of viral assembly. The E protein participates in viral assembly and budding, whereas the highly hydrophobic N protein presumably is the only structural protein that directly binds to the RNA genome.

COVID‐19 is typically accompanied by fever, cough, fatigue, and difficulty of breathing; however mild or asymptomatic courses and other symptoms, like nasal congestion, loss of taste and smell, and diarrhea have been reported (14). Given the global health burden of COVID-19, the development of vaccines that efficiently stimulate immunity in naive individuals is urgently required. From experience with other human pathogenic coronaviruses, including SARS-CoV-1 and MERS-CoV, it was deduced that the structural proteins of SARS-CoV-2 are prime candidates of antiviral immune responses, including neutralizing antibody responses. The SARS-CoV-2 S protein has been shown to induce neutralizing antibodies and has been chosen as the prime antigen of recently established COVID-19 vaccine formulations (15, 16).

*In silico* prediction of B cell epitopes was employed for the structural proteins, including S and M proteins (17–22). The immunogenicity and immunodominance of such epitopes is crucial for vaccine development to induce immunity to SARS-CoV-2 (23). The M protein may also harbor B cell epitopes that can mount a neutralizing antibody response as suggested for SARS-CoV-1 (24, 25). From broad mapping studies using antibody samples of COVID-19 patients, B cell epitopes located in the S and N protein were shown to be immunodominant (21, 26, 27). However, epitopes of SARS-CoV-2 M protein recognized by COVID-19 patients in the acute and convalescent phase of the disease are less studied. In order to determine immunodominant epitopes of SARS-CoV-2 M protein, we used a set of overlapping peptides. Plasma samples were derived from the acute and convalescent phase of COVID-19 at time points with different post-illness onset (PIO). Antibodies were also characterized using immunodominant peptides derived from the N and S protein (21) as well as from an *E. coli* expressed fusion protein harboring major portions of RBD.

## Material and Methods

### Patients

Blood samples were obtained in accordance with the ethics committee of the University Hospital Münster (local ethics committee approval: AZ 2020-220-f-S). Participants were informed regarding the research project and written informed consent was obtained from all participants before enrollment. COVID-19 patients were enrolled between April and November 2020. Diagnosis followed the clinical description released by the World Health Organization (14). COVID‐19 patients were confirmed by the use of SARS-CoV‐2-specific RT‐PCR analysis of oropharyngeal and nasopharyngeal swabs (28). Patients having acute disease (n = 32) were hospitalized in the university clinic, including interventions at ICU (n = 15). Plasma from the convalescent phase of infection was obtained from COVID-19 patients during visits in the outpatient care unit of the hospital (29). Convalescent patients (n = 30) did not show typical symptoms at presentation and experienced a prior two weeks quarantine at home. Commercial SARS-CoV‐2-specific antibody assays used in the routine diagnosis of COVID-19 at the University Hospital Münster (Abbott SARS-CoV-2 IgG assay, CMIA, Architect) were employed (**Supplemental Tables 1** and **2**). Plasma samples used for the control group were from 44 different healthy individuals and banked from board-approved observational study protocols of the hospital between June 2017 and July 2019. Biological samples from all patients were blindly selected with no considerations for age or sex.

### Peptides

SARS-CoV-2 sequences spanning the membrane (M) gene were obtained from NCBI GenBank accession number MN908947.3. Approximately 21 N-terminal biotinylated 20-mer peptides having overlapping sequences of 10 residues were synthesized (Peptide Speciality Laboratories GmbH, Heidelberg). Peptide S and peptide N were synthesized accordingly and contained sequences as reported previously (21). Lyophilized peptides were dissolved in DMSO (Carl Roth GmbH und Co KG, Cat. No. 4720.1) and PBS (Sigma-Aldrich, Cat. No. D8537) to obtain stock solutions of 0.5 to 1 mg/ml. A peptide with a random amino acid sequence (20-mer) served as negative control (data not shown).

### ELISA

Pre-blocked streptavidin-coated ELISA plates (Greiner bio one, Cat. No. 655990) were incubated with peptides at a final concentration of 1 µg/ml in PBS for 2 h at room temperature (RT). Patient plasma was diluted at 1:250 in PowerBlock (0.1-fold pre-diluted in PBS from a 10-fold stock solution; Biogenex Laboratories, Cat. No. HK083-50K). Plasma samples were incubated overnight at RT. Horseradish peroxidase (HRP)-conjugated polyclonal goat antiserum was used that is directed to the constant region (Fc) of all four human immunoglobulin (IgG) subclasses (BioRad Laboratories, Cat. No. AHP1323P). HRP-conjugated polyclonal goat antiserum was used for detection of the μ-chain of human IgM (Sigma-Aldrich, Cat. No. A6907-1ML). Antibody incubation was performed at a dilution of 1:10,000 (IgG) and 1:5,000 (IgM) for 2 h. All washing steps (five times) were performed using PBS/0.05% Tween-20 (AppliChem, A4974, 0250). Tetramethylbenzidine (TMB) substrate (BD Biosciences, Cat. No. 55214) was incubated for 20 min and reaction was stopped by the addition of 2N sulfuric acid (AppliChem, Cat. No. 182105.1208). Absorbance was measured at 450 nm using an Infinite M200 plate reader (Tecan, Switzerland). OD values from a control well without peptide were used for each plasma sample and obtained values were subtracted from background OD. Repeated measurements of identical probes resulted in a low variance (mean 0.08 ± 0.01). A cut-off for positivity was set as the mean value plus two standard deviations (2SD) obtained with control sera.

### Bacterial Expression of SARS-CoV-2 Spike Protein Fragment

Complementary DNA was generated with 1 µg of RNA from a COVID-19 patient using SuperScript III and hexamers with random sequences according to the protocol of the manufacturer (Invitrogen). Cloning of amino acids S407-579 of the SARS-CoV-2 spike protein was performed by using Platinum SuperFI II Tag-polymerase (Invitrogen) and primers

5’-GG**CCATGG** TCAGACAAATCGCTCCAGGG-3’/

5’-GC**GGATCC**TCAGTGGTGGTGGTGGTGGTGTGGATCACGGACAGCATCAGTAG-3’ (SARS-CoV-2-specific sequences underlined, restriction enzymes bold) (Eurogentec, Belgium). The resulting 0.5 kb PCR fragment was digested with NcoI and BamHI restriction enzymes (New England Biolabs) and cloned into T7 polymerase regulated *E. coli* expression vector OGWA (30). Expression of his-tagged fusion protein was achieved in *E. coli* BL21 (DE3) (New England Biolabs, UK). Induction was initiated at an OD600 of 0.6–0.8 by addition of 1 mM Isopropyl-β-D-thiogalactopyranosid (IPTG; Thermo Fischer Scientific) at 37°C. Protein purification after lysozyme (Thermo Fischer Scientific) digested bacterial lysates was performed using NI-NTA agarose according to the protocol of the manufacturer (CubeBiotec).

### Western Blot Analysis

The purified protein was separated on a 12.5% SDS polyacrylamide gel and blotted onto a PVDF membrane (Bio-Rad Laboratories). The membrane was blocked for 2 h in Tris-buffered saline (TBS) containing 0.05% Tween 20 (TBS-T, Sigma-Aldrich) and 5% non-fat dry milk (Carl Roth). The membrane was dissected into individual stripes which were incubated with patient plasma (1:250 in 1 ml) overnight at RT. Polyclonal horseradish peroxidase (HRP)-linked secondary antibody (BioRad goat anti-human IgG (Fc):HRP, Cat. No. AHP1323P at 1:10,000 dilution) was incubated for 1 h. As control, an antibody directed against the histidine tag (mouse anti-Penta Histidine Tag : HRP, Bio-Rad Laboratories, clone ABD2.2.20, Cat. No. MCA5995P) was used at 1:1,000 dilution for every blotting experiment. Amersham ECL Western Blotting Detection Reagent (GE Healthcare Life Sciences) was added for staining. Images were obtained using a FUSION SOLO 6S EVOLUTION-CAPT (Vilber, France). Determination of antibody binding was investigated by visual inspection of the blots and by ImageJ (Wayne Rasband, National Institute of Health, USA) analysis. Each experiment contained strips of a positive (anti-histidin antibody) and a negative control (negative control plasma or no plasma). Identical rectangle boxes were identified by anti-histidin positives. The number of pixels within boxes was referenced to the number of pixels in the negative control for each blot (pixel number investigated/pixel number of negative control). A fold value >1.5 was arbitrarily chosen to indicate positives. All positives identified by ImageJ analysis were in agreement with positives identified by visual inspection. Repeated measurements of identical plasma samples did not result in variant results (data not shown).

### Statistical Analyses

Statistical analyses were performed by Kruskal–Wallis 1-way ANOVA and Chi Square tests using SPSS 27 software. Pearson’s correlation coefficient (r), receiver operating characteristic (ROC) analyses, and T test were calculated using GraphPad Prism 6. A *p* value ≤0.05 was chosen to indicate significance. Data are given as mean ± standard deviation (SD).

## Results

### Immunodominant IgG-Specific Epitopes of SARS-CoV-2 M Protein During the Acute and Convalescent Phase of COVID-19

**Figure 1:**
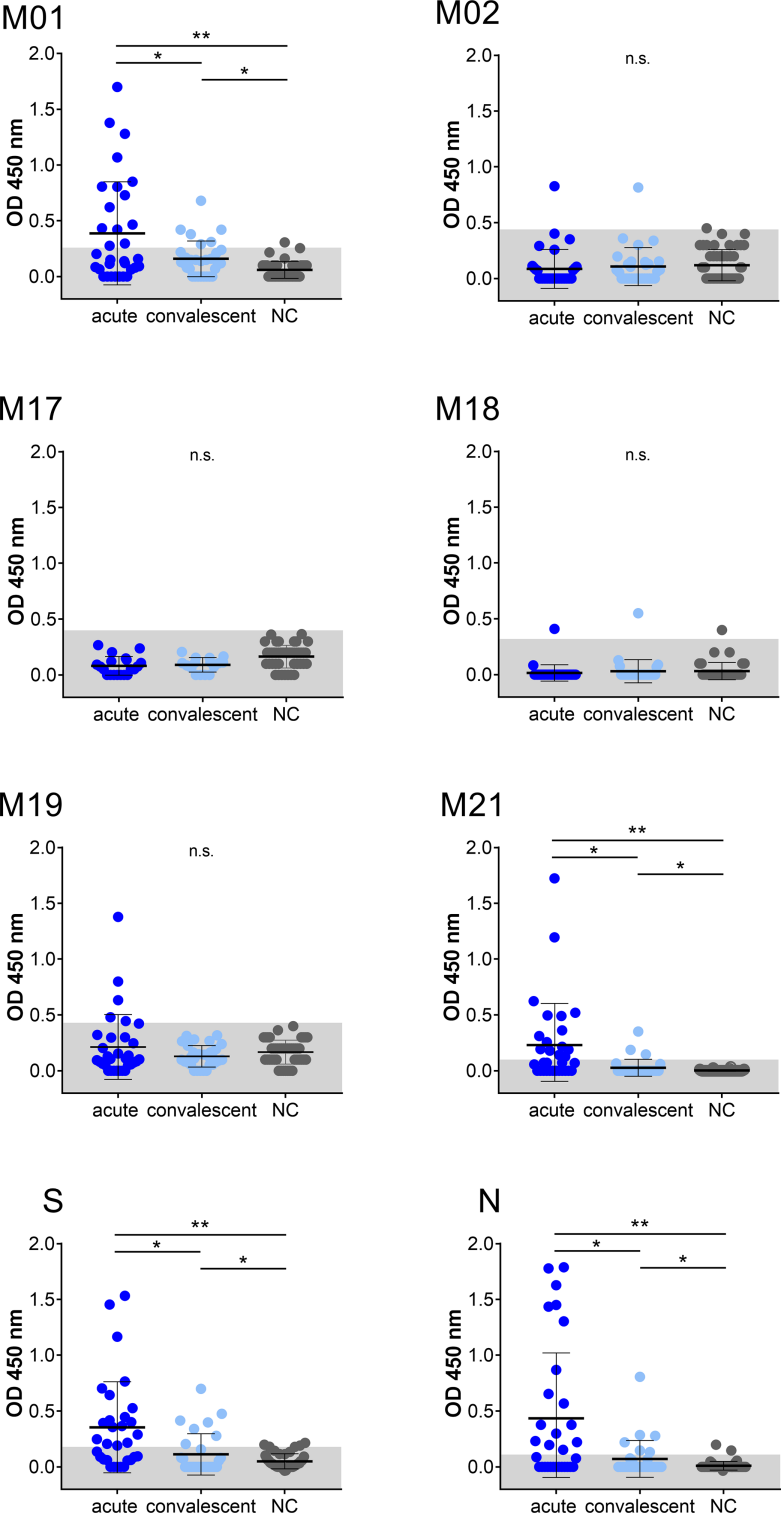
IgG immuno-reactivity obtained with selected peptides of SARS-CoV-2 in two COVID-19 patient cohorts and negative control samples (NC). Each data point represents the OD450 value of one patient. Mean and SD are indicated. The threshold level (mean + 2SD of control) is marked in grey. Data shown is representative of at least 18 independent experiments. Asterisk indicates significance *P < 0.05, **P < 0.0001. n.s., not significant.

Linear B-cell epitopes of SARS-CoV-2 membrane protein were studied during the acute and convalescent phase of COVID-19. Plasma samples were derived from patients following positive SARS-CoV-2 RT-PCR analysis. Approximately 32 plasma samples (S01 to S32) were derived during acute disease while being hospitalized. Approximately 30 plasma samples (R01 to R30) were from COVID-19 patients in the convalescent phase. Major characteristics of COVID-19 patients are presented in **Table 1**. Approximately 44 plasma samples (NC01 to NC44) served as negative controls and were obtained from a historical collection, frozen before the COVID-19 pandemic (mean age 49.4 ± 16 years, range: 19–79 years; 45.5% females and 54.5% males). To characterize immunodominant epitopes of the M protein, a set of 21 peptides having 10 amino acids (aa) overlap was used (**Table 2**). Peptides (M01 to M21) span the complete 222 aa of SARS-CoV-2 M protein. Peptide S and peptide N, previously reported to harbor immunodominant epitopes of the S and N protein of SARS-CoV-2, respectively, were also employed for comparison (21). An IgG-specific ELISA was established using biotin-coupled peptides as coating antigens. Two of the 21 M-specific peptides, peptide M01 and peptide M21, showed the highest IgG-reactivity in COVID-19 patient plasmas (**Figure 1**). Using peptide M01 and M21, 14 (43.8%) and 15 (46.9%) patient samples derived from the acute phase, respectively, were scored positive above thresholds calculated from negative control (mean + 2SD). ROC curve analysis revealed high specificity (>0.98) of the IgG ELISA (**Supplemental Figure 1**). In the convalescent phase, IgG seroconversion of the COVID-19 samples was significantly lower for both peptides (<20.0%). Using peptides M02 and M17-M19 only a few patient samples were scored positive and showed overall low OD450 values (mean OD450 <0.2). Of note, peptide S (56.3%) and peptide N (50.0%) scored positive with plasma samples of the acute phase and showed similar mean OD450 values as observed for peptide M01. However, almost no reactivity (mean OD450 values <0.04 ± 0.08) was observed with the other M-specific peptides which were therefore excluded from further analysis (**Supplemental Figure 2**).

**Table 1:** Characterization of COVID-19 patients.

	Acute	Convalescent
N	32	30
Age (mean/range)	55.7/34–82	42.0/18–64
Male/Female	25/7	28/2
PIO days (mean/range)	18.3/3–40	59.8/28–84

**Table 2:** Peptides of SARS-CoV-2.

Peptide	Sequence	aa
M01	MADSNGTITVEELKKLLEQW	1–20
M02	EELKKLLEQWNLVIGFLFLT	11–30
M03	NLVIGFLFLTWICLLQFAYA	21–40
M04	WICLLQFAYANRNRFLYIIK	31–50
M05	NRNRFLYIIKLIFLWLLWPV	41–60
M06	LIFLWLLWPVTLACFVLAAV	51–70
M07	TLACFVLAAVYRINWITGGI	61–80
M08	YRINWITGGIAIAMACLVGL	71–90
M09	AIAMACLVGLMWLSYFIASF	81–100
M10	MWLSYFIASFRLFARTRSMW	91–110
M11	RLFARTRSMWSFNPETNILL	101–120
M12	SFNPETNILLNVPLHGTILT	111–130
M13	NVPLHGTILTRPLLESELVI	121–140
M14	RPLLESELVIGAVILRGHLR	131–150
M15	GAVILRGHLRIAGHHLGRCD	141–160
M16	IAGHHLGRCDIKDLPKEITV	151–170
M17	IKDLPKEITVATSRTLSYYK	161–180
M18	ATSRTLSYYKLGASQRVAGD	171–190
M19	LGASQRVAGDSGFAAYSRYR	181–200
M20	SGFAAYSRYRIGNYKLNTDH	191–210
M21	IGNYKLNTDHSSSSDNIALLVQ	201–222
S	TESNKKFLPFQQFGRDIA	553–570
N	NNAAIVLQLPQGTTLPKG	153–170

### IgM-Specific Antibody Response to Immunodominant Epitopes of M Protein

**Figure 2:**
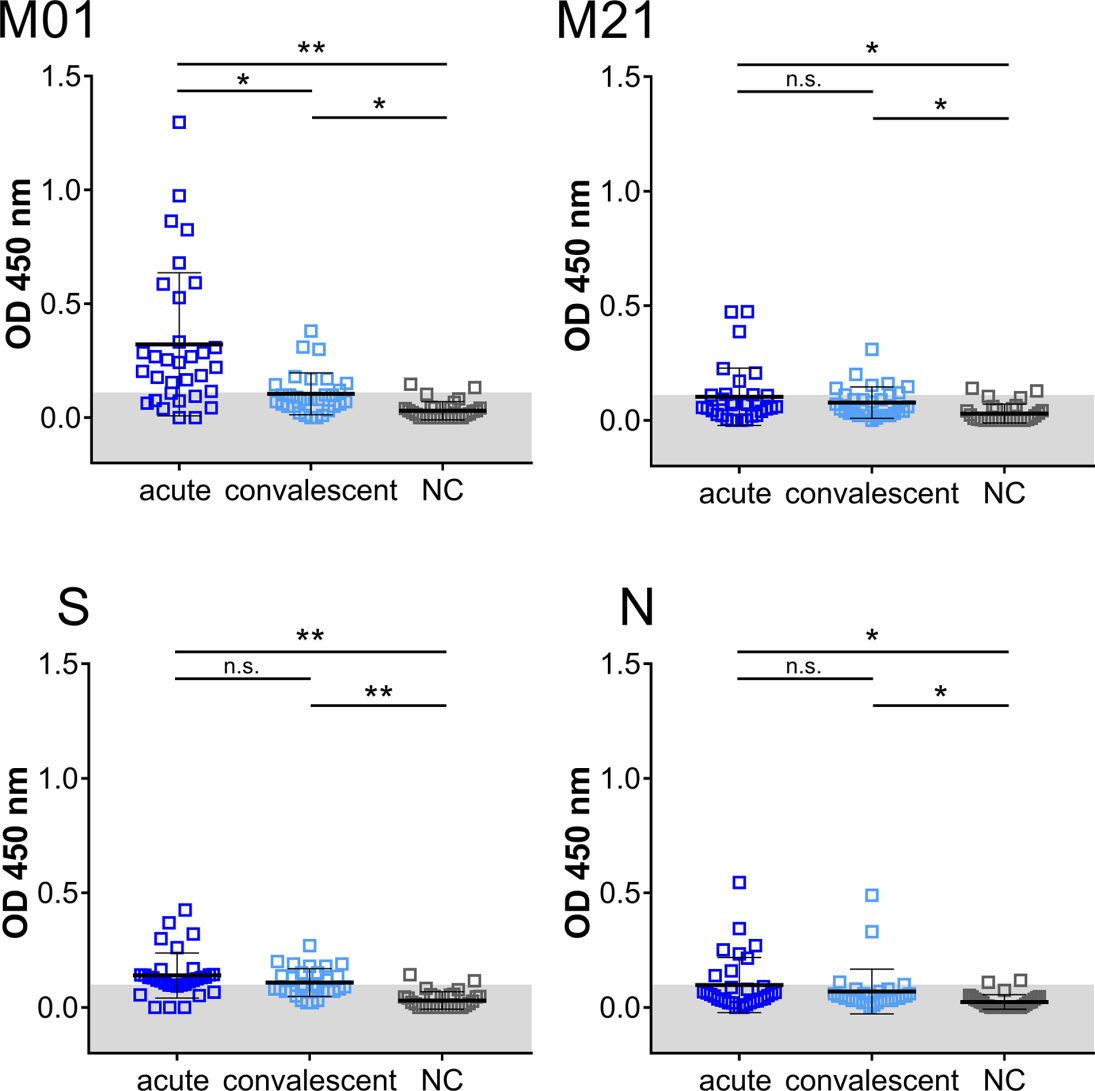
IgM immuno-reactivity obtained with immunodominant peptides in the acute and convalescent phase and negative control group (NC). Each data point represents the OD450 value of one patient. Mean and SD are indicated. The threshold level (mean + 2SD of control) is marked in gray. Data shown is representative of five independent experiments. Asterisk indicates significance *P < 0.05, **P < 0.0001. n.s., not significant.

Having established that peptides M01 and M21 revealed high IgG-specific reactivity, the IgM-specific response was also investigated (**Figure 2**). In the acute phase, 71.9% (peptide M01) and 25.0% (peptide M21) of patient samples were scored positive (>mean + 2SD of control) with mean OD450 values of 0.3 ± 0.3 and 0.1 ± 0.1, respectively. Of note, seropositivity of peptide S (68.8%) and peptide N (25.0%) in the acute phase was in the same range as observed for peptide M01 although significantly lower mean OD450 values were observed for peptide S (0.1 ± 0.09). In the convalescent phase, IgM-specific seropositivity using peptide M01 was lower (30.0%) as compared to the acute phase, while values obtained with peptide M21 (23.3%) was almost identical to the acute phase. ROC curve analysis revealed high specificity (>0.93) of the IgM ELISA (**Supplemental Figure 3**). Taken together, for IgM- and IgG-specific antibodies mean OD450 values obtained with peptide M01 were in the same range or higher as observed for immunodominant peptides S and N in the acute and convalescent phase (**Supplemental Figure 4**).

### Longitudinal Analysis of Immunodominant Epitopes of SARS-CoV-2 M Protein

**Figure 3:**
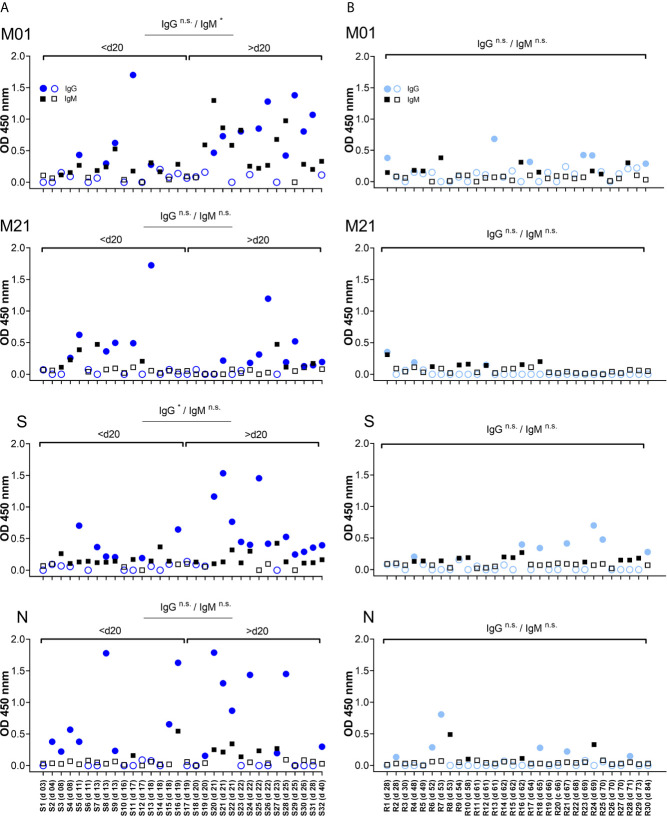
Temporal antibody binding to immunodominant epitopes of SARS-CoV-2. Data are depicted for patients from the acute **(A)** and convalescent phase **(B)**. Plasma samples are arranged according to days PIO (from low to high). Each data point represents the OD450 value observed for one patient and at a given time point PIO. Filled symbols (circle, square) indicate OD450 values above individual threshold. X axis represents COVID-19 patient number (S01-S32 and R01-R30 for acute and convalescent phase, respectively) and days PIO. Significance of the IgG and IgM antibody response is indicated for time periods before/after day 20 PIO. n.s., not significant.

To follow antibody reactivity at different time points, the IgG- and IgM-specific responses directed to peptide M01 and M21 were analysed with regard to time points PIO. In the acute phase, patient samples were grouped according to day 20 PIO. Overall, a weak correlation coefficient (r <0.48) between OD value and day PIO was observed for peptide M01 (**Supplemental Figure 5**). For the IgM-specific response, only peptide M01 showed significantly increased OD450 values following days 20 PIO (**Figure 3**). For the IgG-specific response, peptide S singularly showed significantly increased OD450 values before day 20 PIO. For peptide M21 and peptide N, the IgM- and IgG-specific antibody response did not change between time periods. Of note, for convalescent plasma samples the longitudinal analysis of IgM- and IgG-specific responses did not indicate significance regardless of time periods analysed suggesting that at this phase of COVID-19 the magnitude of the antibody responses is heterogeneous.

### Antibody Response to *E. coli* Expressed Spike Fragment

In order to extend our findings of the acute and convalescent antibody response, the RBD domain of the spike protein was included as an antigen. The RBD domain of the S protein has been proposed to be of high importance for induction of immunity against COVID-19 (31). Using a bacterial expression system, amino acids S407-579 of SARS-CoV-2 spike protein were expressed, including a major portion of the RBD domain fused to a C-terminal poly-histidin tag (**Supplemental Figures 6A, B**). Bacterial expression gave rise to a protein band (S407-579his) of ~20 kd that was recognized by anti-histidin antibody (**Supplemental Figure 6C**). Using Western blot analyses, none of the 44 control plasma samples resulted in a positive IgG-specific binding to the fusion protein. However, plasma samples from COVID-19 patients were scored positive to bind to the S407-579his fusion protein (**Supplemental Figure 7**). In summary, significantly more patients from the acute (50.0%) compared to the convalescent phase (23.3%) were scored positive to contain antibodies against S407-579his fusion protein (**Supplemental Tables 1** and **2**). In the acute phase, number of patients having antibodies against S407-579his fusion protein was almost similar with regard to days PIO (35.3% positives at days <20 PIO and 66.6% at days 20 PIO, respectively). In the convalescent phase, all anti-407-579his positives were recorded at day >65 PIO.

### Detection of SARS-CoV-2 Using Combined Linear Epitopes

Detection of SARS-CoV-2 antibodies is an important tool for diagnosis and a combination of different antigens may increase sensitivity. On an individual patient basis, similar portions of patients from the acute (84.4%) and convalescent phase (63.3%) had IgG-specific antibodies that recognized either one of the four peptides (peptides M01, M21, S and N) and/or S407-579his fusion protein, individually or mixed (**Table 3**). The IgM-specific response to the four peptides only marginally increased overall sensitivity as compared to the IgG response (<87.5 and <84.4%, respectively) and was omitted from further analysis. Inclusion of fusion protein S407-579his as antigen (<84.4%) did not significantly increase detection rates achieved by the four peptides alone (<81.3%). However, when antibody response to peptide S was absent, a higher number of patients (<10%) could be detected by S407-579his fusion protein alone. Of note, a moderate number of COVID-19 patients (<16.7%) could be detected by the two M-specific peptides alone (when antibodies to peptides S and N were absent) suggesting that the M-specific antibody response may add to the overall detection rate. Using fusion protein and peptide S alone for antibody detection, a significantly increased number of patients were scored positive in the acute (43.8%) as compared to the convalescent phase (13.3%). In patients that had antibodies to all four peptides, a higher portion was observed for the acute (15.6%) compared to the convalescent phase (0%) indicating an overall broader antibody response at early time points of disease.

**Table 3:** IgG antibody response against combined SARS-CoV-2 antigens.

Antigen	Acute N (%)	Convalescent N (%)
M01, M21, S, N, S407-579his	27 (84.4) ^n.s.^	19 (63.3)
M01, M21, S, N	26 (81.3)*	16 (53.3)
S407-579his (S negative)	2 (6.2) ^n.s.^	3 (10.0)
M01, M21 (S + N negative)	2 (6.2) ^n.s.^	5 (16.7)
S407-579his + S	14 (43.8)*	4 (13.3)
M01 + M21 +S + N	5 (15.6)*	0 (0)

*Indicates significance (p > 0.05) of acute versus convalescent using Chi-squared test; n.s., not significant.

## Discussion

Serological assays are important to determine the exposure to SARS-CoV-2 in the general population and to gain knowledge about the temporal recognition of epitopes during COVID-19. The immune response directed to the virus spike protein has raised much interest, since this protein directs major arms of the immune system, e.g. by induction of neutralizing antibodies (15, 16). However, limited information about the properties of the SARS CoV-2 membrane protein is available. To our knowledge, a detailed characterization of immunodominant B-cell epitopes in the membrane protein of SARS CoV-2 during the acute and convalescent phase of COVID-19 is compared here for the first time. As the M protein of coronaviruses represents the most abundant protein of the viral particle, a presumably high level of antibody reactivity can be expected during SARS-CoV-2 infection. Our data suggest that the level of antibody response recognizing linear epitopes of the M protein is in the same range as for epitopes of other structural viral proteins, namely, the spike protein and the nucleocapsid protein corroborating that the M protein is highly immunogenic in COVID-19 patients.

Previously, using a broad epitope mapping of SARS-CoV-2 proteins including the M protein, it was revealed that the primary antibody response of COVID-19 patients is directed to the S and N protein (21, 26, 27). Using phage display experiments, a dominant IgG- and IgA-specific antibody response to peptides of S and N was identified, while antibodies against M were only occasionally detected in samples of COVID-19 patients (27). In a peptide-based mapping analyses reported by Amrun and colleagues, using overlapping 18-mer peptides, none of the M-specific peptides was recognized by antibodies of COVID-19 patients when a lower plasma dilution (1:1,000) and peptide pools (five to eight peptides) were used (21). In a COVID-19 patient cohort early after infection (around day 3 PIO), the IgM- and IgG-specific antibody response was studied using a library of 15 amino acid long peptides (26). Five epitopes residing in the M-protein could be identified, however, only a few samples of altogether ten patients scored positive. Our approach dissected the antibody response to linear B cell epitopes by the use of 20-mer overlapping peptides in two patient cohorts of COVID-19 patients at about 18 and 60 days PIO. From our panel of 21 peptides only two peptides, stemming from the very N- and C-terminal ends of the M protein, peptides M01 and M21, were specifically recognized by antibodies of COVID-19 patients, while the other peptides did not provoke specific antibody reactivity as compared to historical plasma samples collected before the pandemic. In contrast to other peptide-based B-cell epitope mapping analyses of the coronavirus M protein (19, 32, 33), we and others employed the biotin–streptavidin system for peptide coating (21, 26, 32). The proposed topology of the M protein may however elucidate the observed antibody reactivity. In addition, *in silico* prediction of B cell epitopes also suggested various epitopes, including the N- and C-terminus of M protein (19, 22, 34). The N-terminus (aa 1 to ~14) is proposed to be exposed to the exterior of the virus, while the long C-terminal portion of M (following aa ~85–99), representing about half of the amino acid residues, is proposed to be inside the viral particle (7–9). Epitopes residing within the N-terminal end of SARS-CoV-2 have also been observed by others (26, 33, 35). The N-terminus of the M protein contains the only N-glycosylation site of the protein, a highly conserved residue among coronaviruses. N-glycosylation however may not affect Golgi-based sorting (9). The N-terminal part of the M protein at the exterior of the viral coronavirus envelope is followed by hydrophobic stretches that contain three putative transmembrane helices, all of which may be less exposed to B cells (7). A peptide encoding aa 196–200 was recognized by IgG- and IgM-specific antibodies in a minor portion of COVID-19 patients; however respective peptides (M19 and M20) bound very low antibodies in our study (26). Amino acids 195–210 were reported to harbor a B cell epitope as determined by an immortalized monoclonal antibody of a SARS CoV-1 convalescent patient (9) corroborating our results obtained with peptide M21 located at the C-terminal end of SARS-CoV-2. However, as compared to B-cell epitope mapping analyses of SARS-CoV-1, the middle portion of the cytoplasmic tail (aa 132–186) rather than the very C-terminus showed highest antibody binding suggesting that the response to linear epitopes of the M protein may differ between human coronavirus members (32). In addition, amino acids 183–197 and amino acids 176–180 showed antibody reactivity in COVID-19 patients, while this region did not result in significant binding above background in our assays (19, 26). In summary, while differences of the experimental methodology and/or patient cohorts might account for the discrepancy of findings on the characterization of immunodominant epitopes of SARS-CoV-2, our data indicate that during the first weeks of COVID-19 an IgM- and IgG-specific antibody response is raised in a significant portion of COVID-19 patients to the N- and C-terminal ends of the M protein with almost identical levels as observed for epitopes located in the S and N protein.

Anti-SARS-CoV-2 antibodies were reported to be detected as early as 3–4 days PIO (36). In our analyses covering COVID-19 patients from the acute phase, a significant portion of patients had IgM and IgG antibodies against peptide M01 before day 20 PIO, with the earliest detection at day 8 PIO. Of note, the IgM antibody reactivity directed against peptide M01 was found to be increased following day 20 PIO, suggesting that the N-terminus is increasingly stimulating an IgM B cell response during acute disease. Peptide M21 was recognized by fewer patients in the acute phase suggesting that this epitope is less immunogenic. Although reactivities of antibodies to peptides M01, M21, S and N were altogether lower in the convalescent phase of COVID-19, a portion of the patients contained antibodies against peptide M01 at this phase suggesting that antibodies directed to the N-terminus of the M protein may persist in the convalescent phase, however at lower levels.

It was concluded that the level of antibodies to linear epitopes of SARS-CoV-2 proteins, including peptides S and N, correlates to the severity of the disease (21). Our study has limitations. Consecutive time points from the same patient were not assessed and convalescent patients, not admitted to hospitalization, have a likely milder course of disease. In addition, all 62 COVID-19 samples were derived from single institution only. Epitopes within peptide 20-mers were not further sub-characterized to identify critical amino acids. Notwithstanding, the antibody reactivity against peptide M01 was in the same range as directed to immunodominant epitopes present in peptides S and N in both cohorts suggesting that similar antibody responses to all SARS-CoV-2 structural proteins are mounted in the two phases of COVID-19.

Antibodies to several B cell epitopes of the spike protein were identified to induce neutralizing antibodies (37–39). RBD was characterized to harbor major epitopes of the neutralizing antibody response. We also assessed antibody binding to a fusion protein containing major parts of the RBD region in the two COVID-19 patient cohorts. As also observed in our B cell epitope mapping analysis, a much higher rate of antibody detection was found for the RBD fusion protein in the acute as compared to the convalescent phase. Of note, plasma samples from convalescent patients have been used for therapy of COVID-19 and many current vaccines include RBD (15, 16, 40). Based on mostly theoretical and *in silico* analyses, M protein of SARS-CoV-2 was also suggested to represent a candidate of vaccines (41). Whether antibodies directed to the M protein can neutralize virus as suggested for SARS-CoV-1 is not known (24–26). As peptides M01 and M21 are located in two highly conserved regions of the SARS-CoV-2 genome, such antibodies may evade the higher variations observed for other epitopes of SARS-CoV-2 structural proteins.

## Data Availability Statement

The raw data supporting the conclusions of this article will be made available by the authors, without undue reservation.

## Ethics Statement

The studies involving human participants were reviewed and approved by ethics committee of the University Hospital Münster. The patients/participants provided their written informed consent to participate in this study.

## Author Contributions

AZ and HS contributed to conception, design and supervision of the study. P-RT, RV, IS, KO, and CF organized the database. PJ, PS, and MW performed experiments. PJ, PS, MW, and AZ analyzed data. PJ, PS, and AZ wrote sections of the manuscript. All authors contributed to the article and approved the submitted version.

## Conflict of Interest

The authors declare that the research was conducted in the absence of any commercial or financial relationships that could be construed as a potential conflict of interest.

## Publisher’s Note

All claims expressed in this article are solely those of the authors and do not necessarily represent those of their affiliated organizations, or those of the publisher, the editors and the reviewers. Any product that may be evaluated in this article, or claim that may be made by its manufacturer, is not guaranteed or endorsed by the publisher.

## Abbreviations

aa, amino acid; E, small membrane protein; ELISA, enzyme-linked immunosorbent assay; IgG, human immunoglobulin G; ICU, intensive care unit; M, membrane protein; MERS-CoV, middle-east respiratory syndrome coronavirus; N, nucleocapsid protein; OD, optical density; PIO, post-illness onset; RBD, receptor binding domain; S, spike protein; SARS-CoV-1/2, severe acute respiratory syndrome coronavirus ½.
