# Supplementary material for: Antibody Response to SARS-CoV-2 Membrane Protein in Patients of the Acute and Convalescent Phase of COVID-19
Source: Front Immunol. 2021 Aug 4;12:679841. doi: 10.3389/fimmu.2021.679841 (PMC8371319; doi:10.3389/fimmu.2021.679841)
Supplement: Supplementary file 1 [file DataSheet_1.docx]

**SUPPLEMENTAL DATA**

**Antibody response to SARS-CoV-2 membrane protein in PATIENTS OF the acute and convalescent phase of COVID-19**

Philipp Jörrißen*, Paula Schütz*, Matthias Weiand, Richard Vollenberg, Inga Marie Schrempf, Kevin Ochs, Christopher Frömmel, Phil-Robin Tepasse, Hartmut Schmidt, Andree Zibert^1^

Medizinische Klinik B, Universitätsklinikum Münster, Münster, Germany

*equal contribution

^1^Correspondence to: Medizinische Klinik B (Gastroenterologie, Hepatologie, Endokrinologie, Klinische Infektiologie), Universitätsklinikum Münster, Albert-Schweitzer-Campus 1, Gebäude A14, D-48149 Münster, Germany.

Email: andree.zibert@ukmuenster.de

Telephone: +49-251- 83-57935 Fax: +49-251 83-57771

**Supplemental Table 1**: Patient-specific analysis during acute phase of COVID-19

|  |  |  |  | peptides^#^ | | |  | | fusion protein |
| --- | --- | --- | --- | --- | --- | --- | --- | --- | --- |
| Patient | Age | PIO | Ab | M01 | M21 | S | N |  | S407-579his |
| S01 | 65 | 3 | nd | -/- | -/- | -/- | -/- |  | neg |
| S02 | 37 | 4 | nd | -/- | -/- | -/- | +/- |  | neg |
| S03 | 34 | 8 | nd | -/+ | -/+ | -/+ | +/- |  | pos |
| S04 | 43 | 8 | pos | -/+ | +/+ | -/+ | +/- |  | neg |
| S05 | 58 | 11 | pos | +/+ | +/+ | +/+ | +/- |  | neg |
| S06 | 53 | 11 | nd | -/- | -/- | -/+ | -/- |  | neg |
| S07 | 57 | 13 | pos | -/+ | -/+ | +/+ | -/- |  | pos |
| S08 | 60 | 13 | pos | +/+ | +/- | +/+ | +/- |  | neg |
| S09 | 51 | 13 | nd | +/+ | +/- | +/+ | +/- |  | pos |
| S10 | 81 | 16 | neg | -/- | -/- | -/- | -/- |  | pos |
| S11 | 58 | 17 | pos | +/+ | +/- | -/+ | -/+ |  | neg |
| S12 | 68 | 17 | nd | -/- | -/+ | +/- | -/- |  | pos |
| S13 | 45 | 18 | pos | +/+ | +/- | -/+ | -/- |  | neg |
| S14 | 63 | 18 | pos | -/+ | -/- | -/+ | -/- |  | neg |
| S15 | 64 | 18 | nd | -/- | -/- | -/+ | +/- |  | neg |
| S16 | 54 | 19 | nd | -/+ | -/- | +/- | +/+ |  | pos |
| S17 | 48 | 19 | nd | -/- | -/- | -/- | -/- |  | neg |
| S18 | 41 | 20 | nd | -/- | -/- | -/+ | -/- |  | neg |
| S19 | 73 | 20 | pos | -/+ | -/- | -/- | +/- |  | neg |
| S20 | 82 | 21 | nd | +/+ | -/- | +/+ | +/+ |  | pos |
| S21 | 57 | 21 | nd | +/+ | +/- | +/+ | +/+ |  | pos |
| S22 | 63 | 21 | nd | -/+ | -/- | +/+ | +/+ |  | neg |
| S23 | 45 | 22 | pos | +/+ | -/- | +/+ | -/+ |  | pos |
| S24 | 71 | 22 | nd | -/+ | +/- | +/+ | +/- |  | pos |
| S25 | 61 | 22 | nd | +/+ | +/- | +/- | -/+ |  | pos |
| S26 | 51 | 22 | pos | +/+ | +/- | +/- | -/- |  | pos |
| S27 | 44 | 23 | pos | -/+ | -/+ | -/+ | +/+ |  | neg |
| S28 | 64 | 25 | pos | +/+ | +/+ | +/+ | +/- |  | pos |
| S29 | 39 | 25 | pos | +/- | +/- | +/- | -/- |  | neg |
| S30 | 49 | 26 | nd | +/+ | +/- | +/+ | -/- |  | pos |
| S31 | 46 | 28 | pos | +/+ | +/+ | +/+ | -/- |  | pos |
| S32 | 57 | 40 | nd | -/+ | +/- | +/+ | +/- |  | pos |
|  |  |  |  |  |  |  |  |  |  |
| Positives % |  |  |  | 43.8/71.9 | 46.9/25.0 | 56.3/68.8 | 50.0/25.0 |  | 50.0 |

^#^Slash divides ELISA results of IgG (left) and IgM seropositivity (right); Ab, commercial SARS-CoV-2 antibody assay; nd, not determined

**Supplemental Table 2**: Patient-specific analysis during convalescent phase of COVID-19

|  |  | |  |  | | peptides^#^ | |  | | fusion protein | |
| --- | --- | --- | --- | --- | --- | --- | --- | --- | --- | --- | --- |
| Patient | Age | PIO | Ab | M01 | M21 | | S | N |  | | S407-579his |
| R01 | 36 | 28 | pos | +/+ | +/+ | | -/- | -/- |  | | neg |
| R02 | 19 | 28 | pos | -/- | -/- | | -/- | +/- |  | | neg |
| R03 | 18 | 30 | pos | -/- | -/- | | -/- | -/- |  | | neg |
| R04 | 63 | 48 | pos | -/+ | +/- | | +/+ | -/- |  | | neg |
| R05 | 64 | 49 | neg | -/+ | -/- | | -/+ | -/- |  | | neg |
| R06 | 61 | 52 | nd | -/- | -/+ | | -/- | +/- |  | | neg |
| R07 | 41 | 53 | neg | -/+ | -/- | | -/+ | +/- |  | | neg |
| R08 | 57 | 53 | nd | -/- | -/- | | -/- | -/+ |  | | neg |
| R09 | 43 | 54 | pos | -/- | -/+ | | -/+ | -/- |  | | neg |
| R10 | 34 | 58 | pos | -/- | -/+ | | -/+ | -/- |  | | neg |
| R11 | 62 | 61 | pos | -/- | -/- | | -/- | -/- |  | | neg |
| R12 | 55 | 61 | pos | -/- | +/+ | | -/- | -/- |  | | neg |
| R13 | 40 | 61 | pos | +/- | -/- | | -/- | -/- |  | | neg |
| R14 | 33 | 62 | neg | -/- | -/- | | -/+ | -/- |  | | neg |
| R15 | 42 | 62 | pos | -/- | -/- | | -/+ | -/- |  | | neg |
| R16 | 56 | 62 | pos | -/+ | -/+ | | +/+ | -/+ |  | | neg |
| R17 | 23 | 64 | pos | +/- | -/- | | -/- | -/- |  | | neg |
| R18 | 51 | 65 | pos | -/+ | -/+ | | +/- | +/- |  | | pos |
| R19 | 29 | 66 | neg | -/- | -/- | | -/- | -/- |  | | neg |
| R20 | 54 | 66 | pos | -/- | -/- | | -/+ | -/- |  | | pos |
| R21 | 24 | 67 | pos | -/- | -/- | | +/- | +/- |  | | pos |
| R22 | 33 | 68 | neg | -/- | -/- | | -/- | -/- |  | | neg |
| R23 | 57 | 69 | neg | +/- | -/- | | -/+ | -/- |  | | neg |
| R24 | 39 | 69 | pos | +/+ | -/- | | +/- | -/+ |  | | pos |
| R25 | 28 | 70 | neg | -/+ | -/- | | +/- | -/- |  | | neg |
| R26 | 51 | 70 | pos | -/- | -/- | | -/- | -/- |  | | pos |
| R27 | 44 | 70 | pos | -/- | -/- | | -/+ | -/- |  | | neg |
| R28 | 33 | 71 | pos | -/+ | -/- | | -/+ | +/- |  | | neg |
| R29 | 38 | 73 | pos | -/- | -/- | | -/+ | -/- |  | | pos |
| R30 | 31 | 84 | pos | +/- | -/- | | +/- | -/- |  | | pos |
|  |  |  |  |  |  | |  |  |  | |  |
| Positives % |  |  |  | 20.0/30.0 | 10.0/23.3 | | 23.3/43.3 | 20.0/10.0 |  | | 23.3 |

^#^Slash divides ELISA results of IgG (left) and IgM seropositivity (right); Ab, commercial SARS-CoV-2 antibody assay; nd, not determined


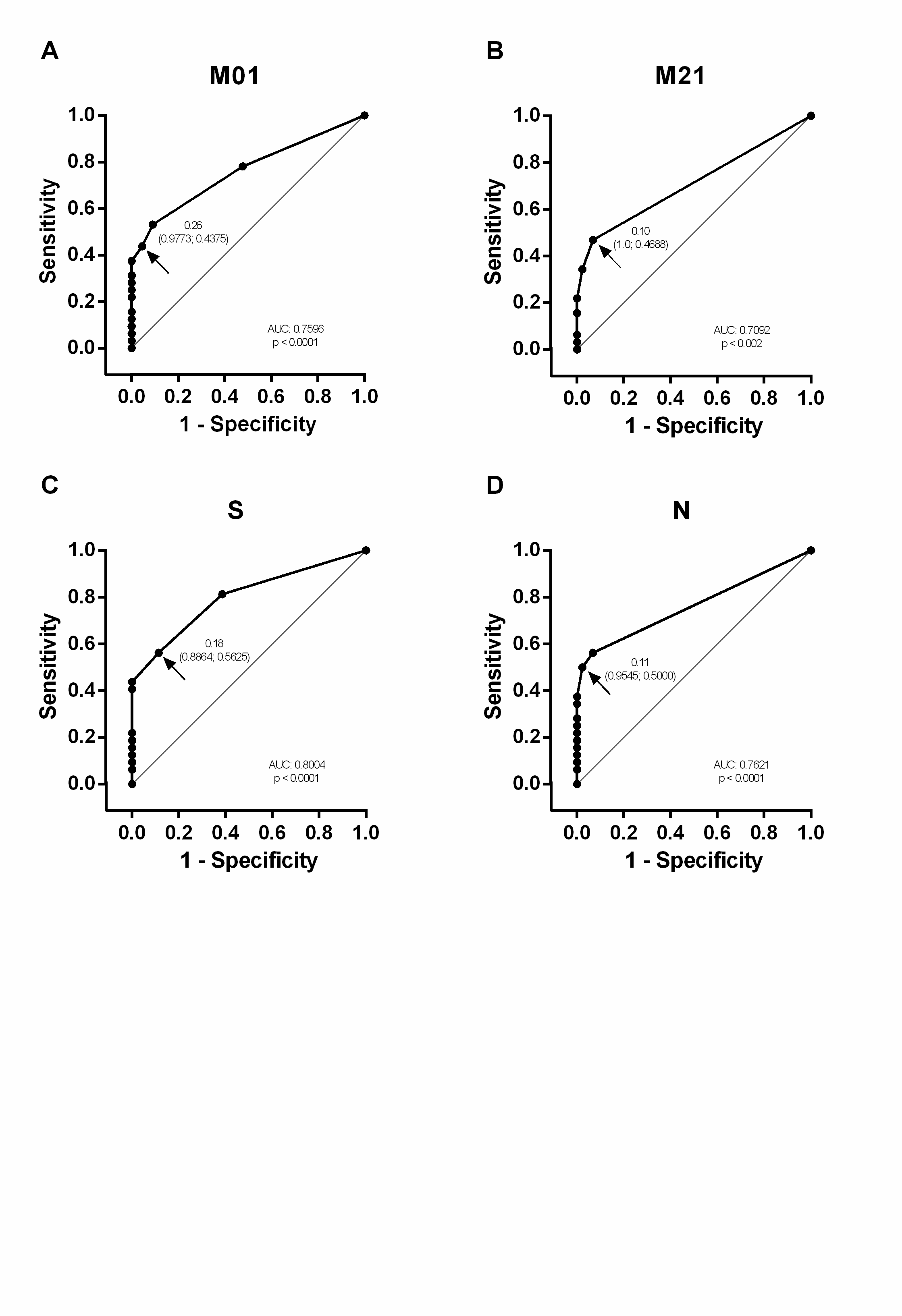


**Supplemental Figure 1: Receiver operating characteristic (ROC) curve analysis of IgG-specific response.** ROC curve analysis of IgG antibody reactivities obtained with peptides M01 (A), M21 (B), S (C) and N (D) is shown for the acute phase. The arrow indicates levels of specificity and sensitivity at threshold (mean+2SD of controls). The area under the curve (AUC) and p values are also given.

**Supplemental Figure**
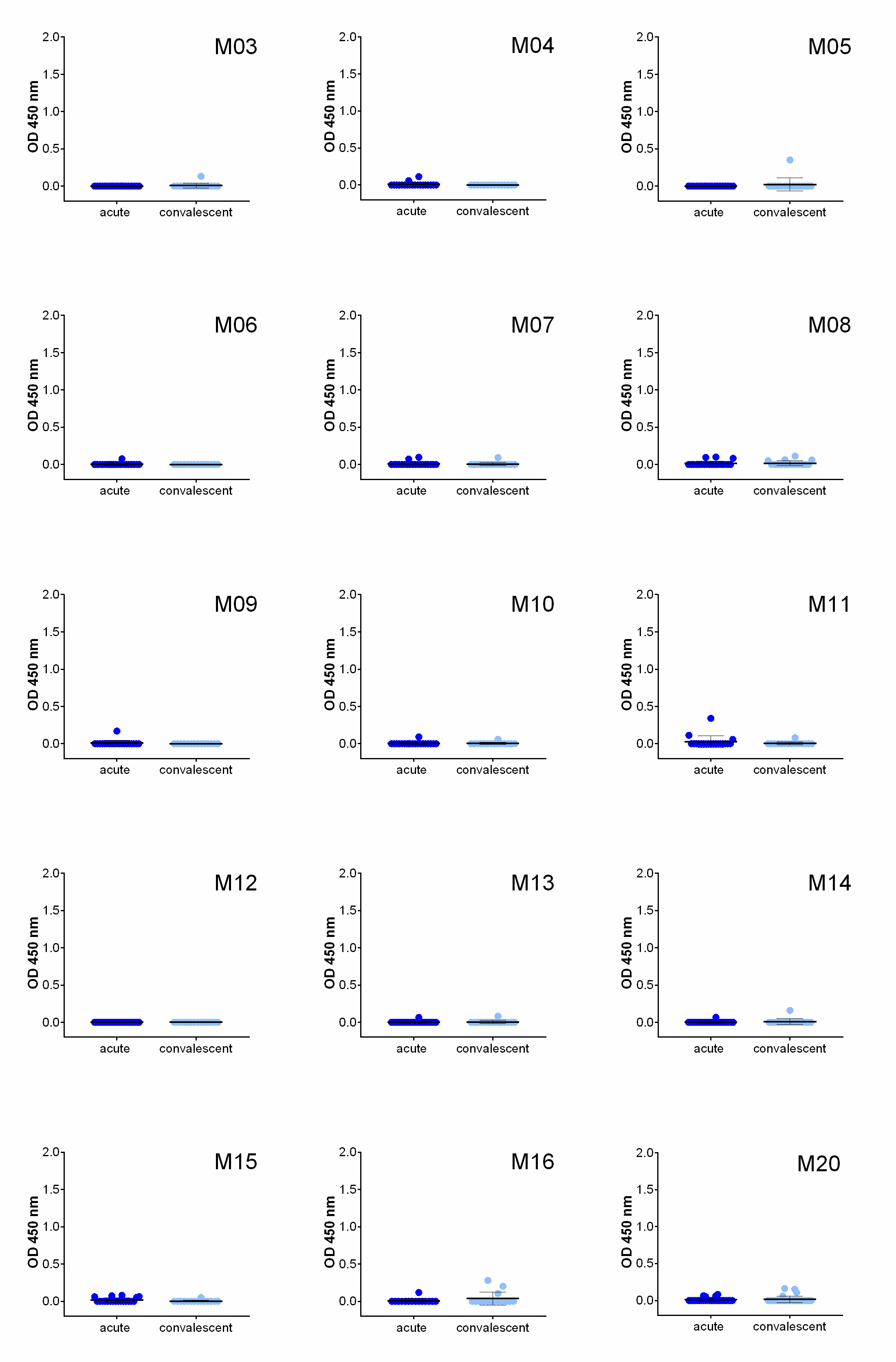
**2:**

IgG-specific reactivity obtained with SARS-CoV-2 M-specific peptides that showed low OD450 values in COVID-19 plasma samples (mean OD450 values <0.04±0.08). Each data point represents the OD450 value of one patient. Mean and SD are indicated. T-test analysis indicated no significance between the acute and convalescent phase for all peptides.


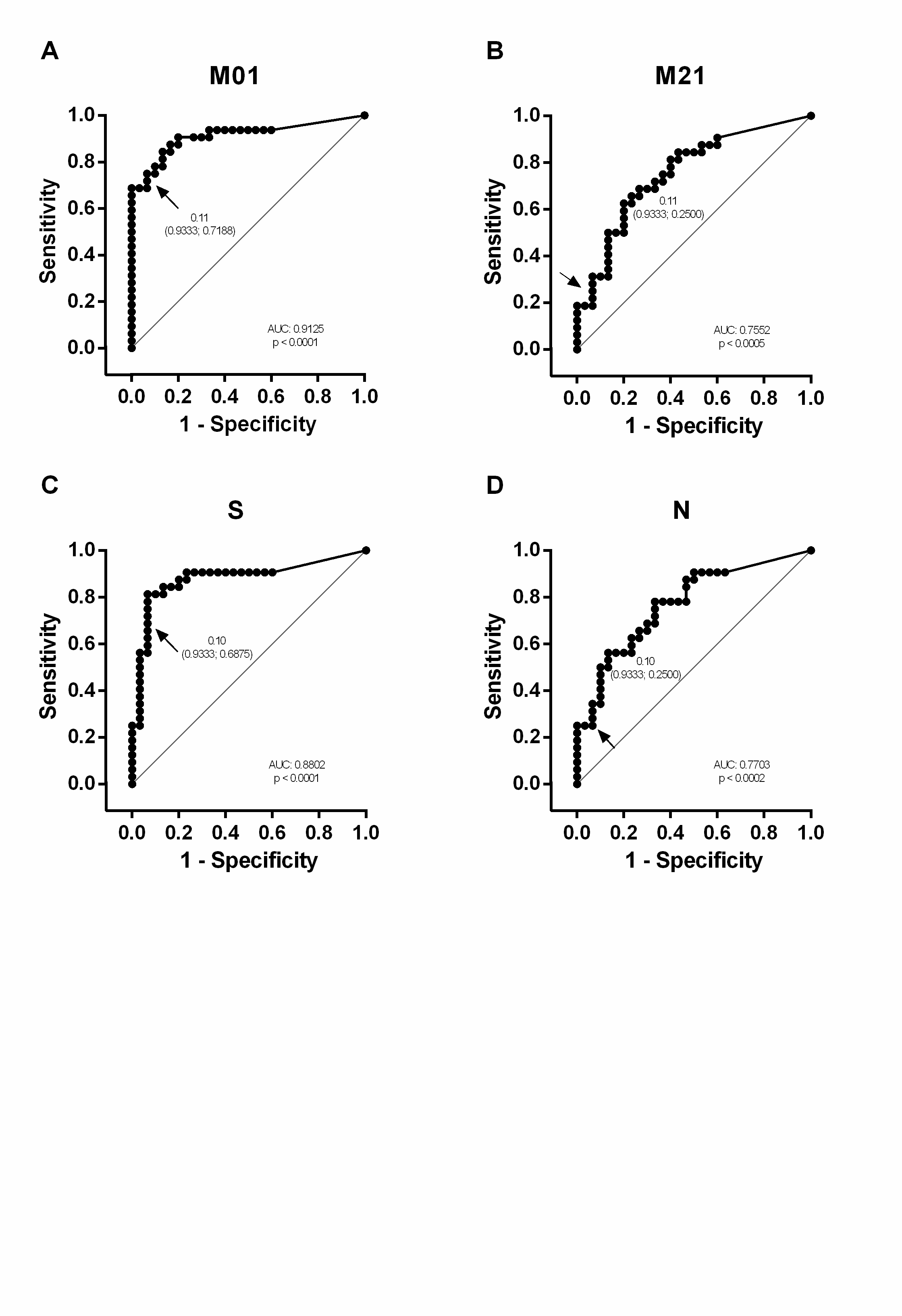


**Supplemental Figure 3:**

**Receiver operating characteristic (ROC) curve analysis.** ROC curve analysis of IgM antibody reactivities obtained with peptides M01 (A), M21 (B), S (C) and N (D) is shown. The arrow indicates levels of specificity and sensitivity at threshold (mean+2SD). The area under the curve (AUC) and p values are also given.


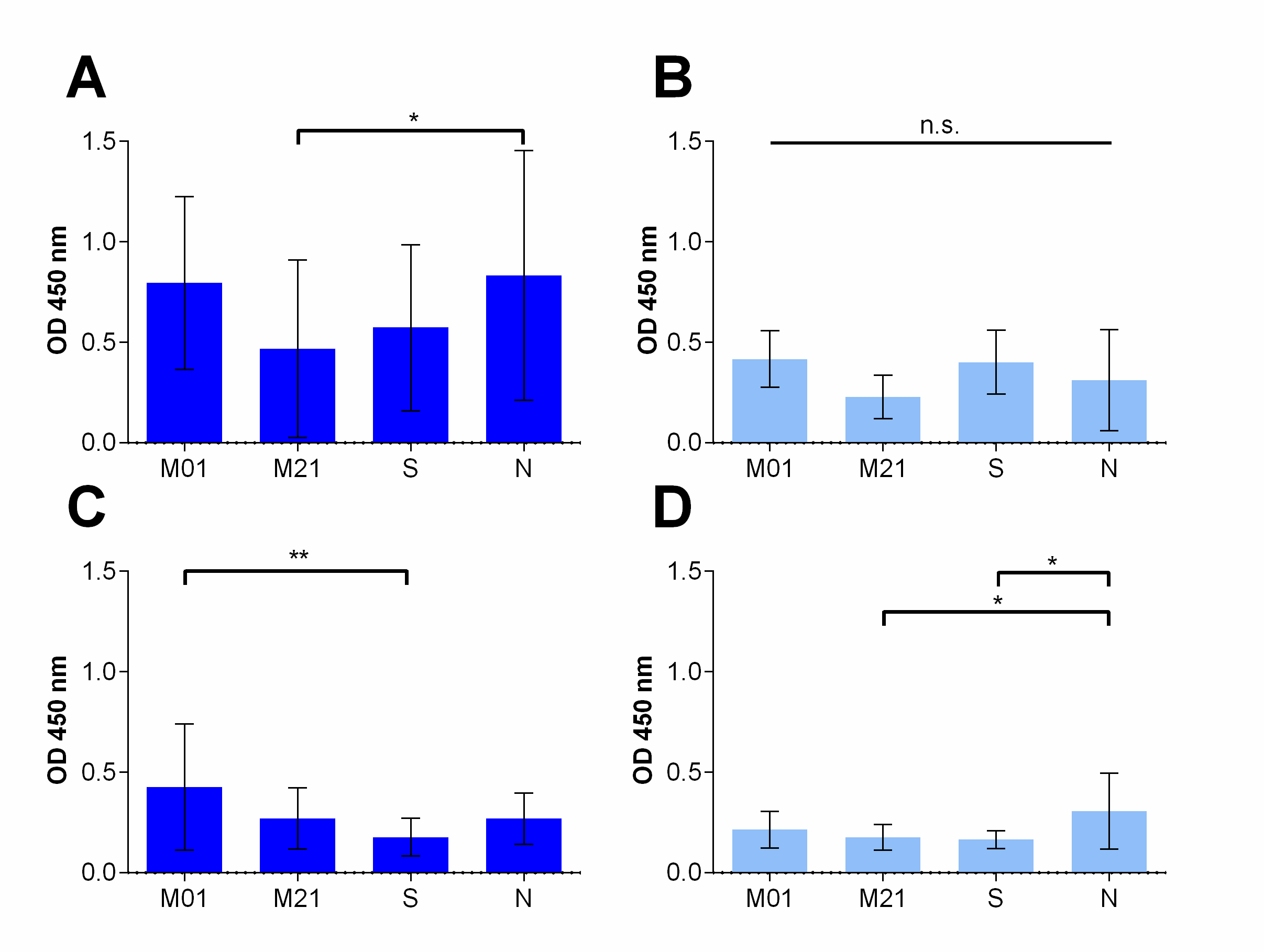


**Supplemental Figure 4: One way analysis of variance (ANOVA) of mean OD450 values.** The IgG- (A and B) and IgM-specific values (C and D) were compared for the acute (A and C) and convalescent phase (B and D). Mean values and SD are indicated. Asterisk indicate where significance levels were observed between peptides. *(P<0.05), **(P<0.0001). n.s., not significant (indicated for comparisons in B and ommitted in the other graphs).


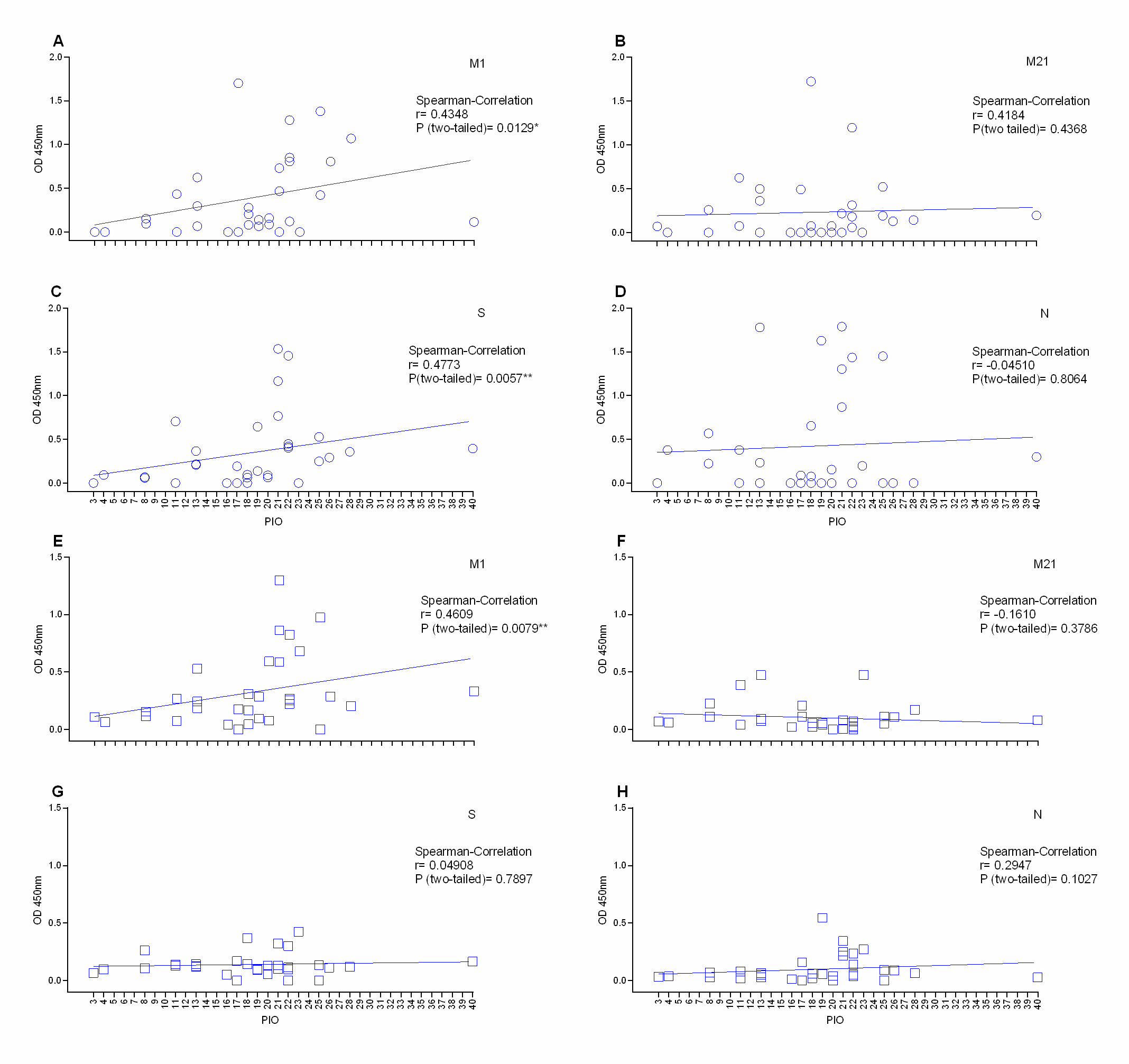


**Supplemental Figure 5: Determination of Pearson’s correlation coefficient**

Correlation coefficients between OD450 value and days PIO was determined for the plasma samples of the acute COVID-19 phase. IgG- (A-D) and IgM-specific reactivities (E-H) were calculated. In the convalescent phase, OD450 values did not reveal a time dependency (data not shown). One circle represents one patient. Pearson’s coefficient and p values are given.

**A**


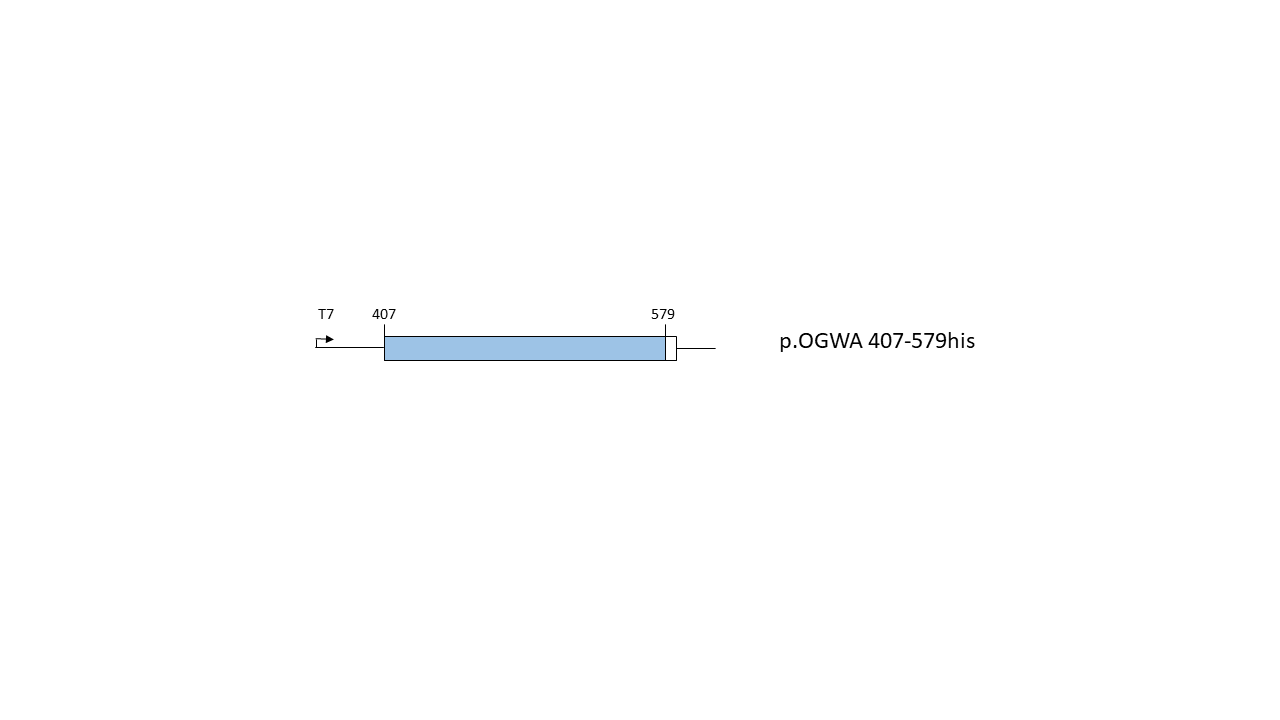


**B**

**M**VRQIAPGQTGKIADYNYKLPDDFTGCVIAWNSNNLDSKVGGNYNYLYRLFRKSNLKPFERDISTEIYQAGSTPCNGVEGFNCYFPLQSYGFQPTNGVGYQPYRVVVLSFELLHAPATVCGPKKSTNLVKNKCVNFNFNGLTGTGVLTESNKKFLPFQQFGRDIADTTDAVRDP**HHHHHHStop**

**C**


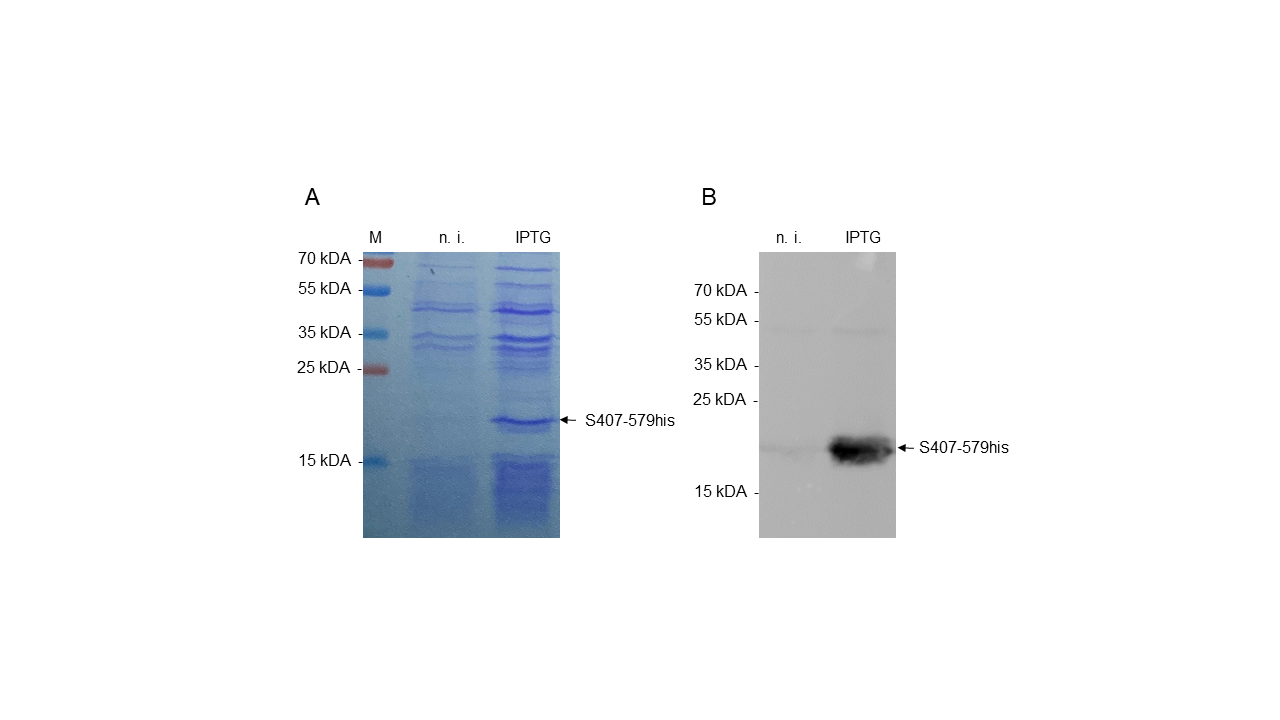


**Supplemental Figure 6: E.coli expression of a spike fusion protein harboring RBD. (A)** Using plasmid p.OGWA407-579his, amino acids S407-579 of SARS-CoV-2 spike protein were expressed, including a major portion of the RBD domain fused to a C-terminal poly-histidin tag. (B) Amino acid sequence of cloned S407-579 (blue). Amino acids from the vector are highlighted in red. (C) Bacterial expression of S407-579his fusion protein revealed a band of ~20 kd recognized by Coomassie Blue staining (left) and by anti-histidin antibody analysis (right). n.i., not induced by IPTG.


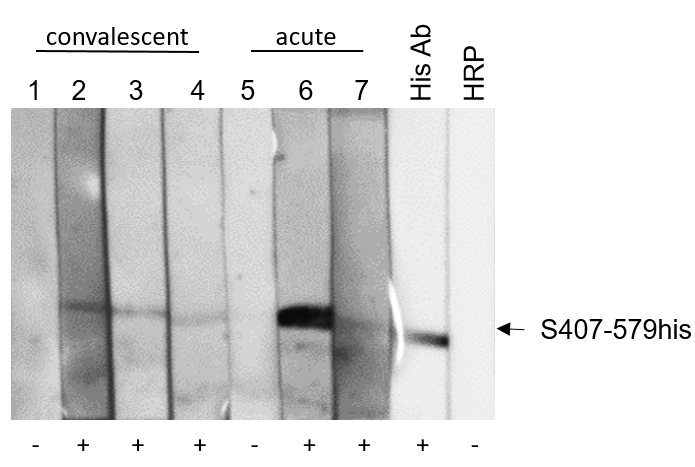


**Supplemental Figure 7:** Western-blot analysis of antibody binding to fusion protein S407-579his. Results obtained with plasma samples from convalescent (lanes 1-4) and acute phase (lanes 5-7) of COVID-19 patients are shown. For control, results from antibody directed to the histidine tag (positive control) and secondary HRP antibody (without addition of plasma sample, negative control) are also given. Results from area quantification experiments are represented by +/- to indicate binding to S407-579his. One experiment of 10 independent experiments is shown.
